# Acute Effects of Different Types of Compression Legwear on Biomechanics of Countermovement Jump: A Statistical Parametric Mapping Analysis

**DOI:** 10.3390/jfmk10030257

**Published:** 2025-07-07

**Authors:** Rui-Feng Huang, Kit-Lun Yick, Qiu-Qiong Shi, Lin Liu, Chu-Hao Li

**Affiliations:** 1 School of Fashion and Textiles, The Hong Kong Polytechnic University, Hong Kong, China; ruifeng.huang@polyu.edu.hk (R.-F.H.); kit-lun.yick@polyu.edu.hk (K.-L.Y.); 13538151863@163.com (L.L.); 24152696r@connect.polyu.hk (C.-H.L.); 2 Engineering Research Center of Sports Health Intelligent Equipment of Hubei Province, Wuhan Sports University, Wuhan 430000, China; 3 Research Institute for Sports Science and Technology, Hong Kong, China; 4 School of Education (Normal School), Dongguan University of Technology, Dongguan 523000, China

**Keywords:** compression garment, joint kinematics, joint kinetics, jump performance, landing biomechanics

## Abstract

**Background**: Compression garments (CG) may influence countermovement jump (CMJ) performance by altering hip and knee biomechanics, but existing evidence remains controversial. This study aimed to compare the effects of compression tights (CTs), compression shorts (CSs), and control shorts (CCs) on CMJ performance and lower-limb biomechanics. **Methods**: Nine physically active men from a university were recruited to perform CMJ while wearing CTs, CSs, and CCs in a randomized sequence for a within-subjects repeated-measures design. A Vicon 3D motion capture system and an AMTI 3D force plate were used to collect biomechanical data. Visual3D software was used to calculate the joint angle, moment, and force of the lower limbs. **Results**: Statistical parametric mapping analysis with repeated measures analysis of variance (ANOVA) revealed that during the propulsion phase of the CMJ, wearing CSs significantly reduced the hip flexion angle compared to wearing CCs (25–36%); meanwhile, wearing CTs significantly reduced the knee extension and flexion moment (34–35%) and decreased the hip extension moment during the propulsion phase (36–37%). In addition, CTs significantly reduced the hip abduction angle during the flight phase (37–39%), and CSs significantly reduced the hip anterior force during the landing phase (59–60%). **Conclusions**: Compression legwear significantly affected the hip and knee biomechanics in propulsion, but these differences were not sufficient to improve the CMJ height. Due to the improvement in hip biomechanics in the flight and landing phases, there may be potential benefits for movement transitions and landing performance in CMJ.

## 1. Introduction

The countermovement jump (CMJ) is a training tool used to evaluate lower-limb muscle strength and measure explosive power and neuromuscular control. CMJ performance is shown to be highly correlated with physical function and in-game performance, including spiking jumps in volleyball, lay-up shot performance in basketball, weightlifting abilities, and the risk of injury caused by differences in landing strategy [1,2,3,4]. Studies have explored the biomechanical determinants that improve CMJ performance (e.g., discrete variables such as jump height and peak joint angle, moment, and force) [5,6,7,8]. However, the results of these studies may have been influenced by the interventions, including the use of specialized training equipment [9,10,11,12].

Compression garments (CGs) have been used in athletic training for decades as supportive training equipment [13]. This is because CGs apply mechanical compression to the body, which may have beneficial effects on the physiology, biomechanics, athletic performance, and proprioception of the users [13,14]. Although most studies have shown that lower-limb CGs have no performance-enhancing effect on jump movements, some researchers have presented contradicting findings [14]. Wannop et al. [15] found that wearing CGs increased CMJ height, while the peak hip flexion angle increased significantly and the peak hip moment was only slightly increased. However, Leabeater et al. [16] presented contradictory results. They found that wearing a CG only had a significant effect on the average eccentric ground reaction force during the CMJ. In addition, de Britto et al. [12] explored CMJ landing performance and found that wearing a CG reduced the range of motion of the knee valgus angle, indicating that CGs can reduce the risk of injury. However, discrepancies in the types of CGs employed across studies indicate a lack of consensus on their influence on CMJ performance, which makes it unclear whether a specific type of CG affects the outcomes [14].

In studies that have investigated the effects of lower-limb CGs on CMJ performance, the main variables are the type of compression legwear, such as compression tights (CTs), compression shorts (CSs), stiff shorts, etc., and the primary biomechanical parameters include joint angles and moments and ground reaction forces (GRFs) [12,15,16]. However, these studies have focused exclusively on peak values, which are referred to as discrete variables or zero-dimensional (0D) variables in statistics. The characteristic of 0D analysis is that data are extracted from the biomechanics–time curves instead of performing statistical analysis on data from the whole movement. Studies have also argued that even though 0D data help simplify statistical analysis compared with the evaluation of continuous curves (i.e., 1D analysis), the use of discrete variables derived from continuous data (i.e., 0D analysis) may obscure movement-specific changes that occur throughout the entire CMJ movement. Statistical parametric mapping (SPM) facilitates the analysis of smooth and continuous data and presents the data in their original and complete state, thereby reducing the possibility of analytical bias due to 0D variable selection. Hughes et al. showed that SPM has an advantage over selecting 0D variables in identifying the biomechanical characteristics of CMJs [17]. However, few comprehensive studies have explored the mechanisms by which legwear affects CMJ biomechanics and performance based on time-continuous data.

Therefore, the purpose of this study was to apply SPM to analyze the differences in biomechanical time curves during CMJ across three legwear conditions and to investigate the underlying biomechanical mechanisms that influence CMJ performance. The conditions included (1) CTs, which provide targeted pressure to the lower-limb muscles, including the hamstring, soleus, vastus, gluteus maximus, and gastrocnemius muscles; (2) CSs, which apply pressure selectively to the above-knee muscles, such as the hamstring, gluteus maximus, and vastus muscles; and (3) control shorts (CC), which are standard sports shorts without engineered compression; these served as the baseline condition. By comparing these conditions, this study aimed to elucidate how variations in compression design affect jump biomechanics. We hypothesized that significant differences would exist in biomechanical time curves during the CMJ across the three legwear conditions (CT, CS, and CC). These differences would reflect distinct biomechanical mechanisms influenced by variations in compression targeting.

## 2. Materials and Methods

### 2.1. Participants

G Power 3.1.9.7 software (Heinrich-Heine-Universität Düsseldorf, Düsseldorf, Germany) was used in this study to calculate the predicted sample size. An effect size of 0.5 was set for the repeated measures ANOVA analysis, with an α of 0.05 and a power of 0.8. The number of groups and measurements were 1 and 3, respectively. The correlation among repeated measures was set at 0.5 and the Nonsphericity Correction ε was set at 1, which yielded a minimum required sample size of 9 participants. Considering potential data loss, 11 physically active males were recruited as subjects through university-wide poster advertisements (age: (mean ± SD) 26.0 ± 3.1 years old; height: 170.2 ± 6.5 cm; body mass: 67.6 ± 8.1 kg; and body mass index: 23.4 ± 3.5 kg/m^2^). All recruited subjects had at least two years lower-limb resistance training experience, and maintained a minimum training frequency of two sessions per week. Before the experiment, all participants completed the physical activity readiness questionnaire [18], confirming their ability to safely perform CMJ. Participants were instructed to abstain from alcohol, caffeine, and smoking for at least 24 h prior to the experiments. All participants were right-leg dominant, and provided written informed consent after being fully informed of the experimental procedures. The study protocol was approved by the university of the first author.

### 2.2. Experimental Protocol

**Figure 1:**
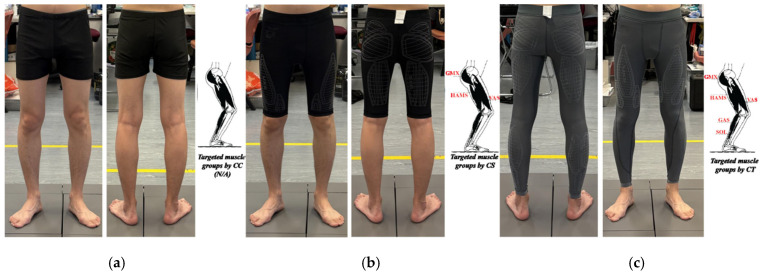
Three types of legwear: (**a**) CC, (**b**) CS, and (**c**) CT.

Three types of commercial legwear were randomly assigned to each participant for testing. The test order was randomized using https://randomizer.org/ (accessed on 15 June 2024). The test conditions are listed in Supplemental File Table S1. The experimental compression legwear (CSs and CTs) was obtained from an Australian brand, 2XU. The three legwear variations are shown in Figure 1, with the inside of the CSs and CTs highlighted to indicate the compression regions and the specific muscle groups that they were designed to support. In Figure 1, HAMS indicates hamstrings, SOL indicates soleus, VAS indicates vastus, GMAX indicates gluteus maximus, and GAS indicates gastrocnemius. The participants were fitted with size-appropriate legwear according to the height and body mass sizing chart provided by the manufacturer.

Prior to testing, all subjects completed a dynamic warm-up that included high knee pulls, Frankenstein warm-up, and forward gate swings for 10 min, followed by stretching for 5 min based on their pretraining routines; they then practiced CMJs in advance, which enabled them to adapt to the position of the force platform and the space of the laboratory, and finally sat quietly for 2 min. Following the Plug-in-Gait Marker Placement protocol in the Vicon Technical Guide, 30 reflective markers were placed on the participants (Figure S1) to construct a lower-limb biomechanical model in Visual3D (v6, C-Motion, Inc., Germantown, MD, USA). Kinematic data were captured using nine infrared cameras (Vicon Motion Systems Ltd., Oxford, UK) sampled at 100 Hz, and synchronized with two AMTI force plates of 50 × 50 cm in dimensions (Advanced Mechanical Technology, Inc., Watertown, MA, USA) to collect kinetic data at a sampling frequency of 1000 Hz.

After system calibration, the participants were required to pose in an anatomical standing position with each foot placed on a separate force plate for two seconds for baseline recording. During testing, participants placed their hands on their waist while performing maximum-effort CMJs according to their natural jumping style. Three successful trials were collected for each condition, with success defined by (1) stable landing without stepping off the plates, (2) continuous marker visibility, and (3) uninterrupted force plate signal acquisition. Failed trials were immediately repeated. Following each condition, participants rested for 15 min before changing to the next type of legwear, with the markers carefully reapplied following the same protocol. This ensured consistent measurement conditions across all test configurations.

### 2.3. Indicators

Based on the biomechanical characteristics of the CMJ and research published on the effects of CGs [12,15,16,19], the following kinetic and kinematic parameters were selected for analysis: vertical ground reaction force (vGRF) [unit: body weight], knee, ankle, and hip joint moments [unit: N∙m/kg], and joint force [unit: N/kg], which were selected as kinetic indicators. The kinematic indicators included the hip, knee and ankle joint angles [unit: deg] and jump height [unit: m]. The definition of the joint coordinate system used in the calculation is shown in Figure S2 [20]. The joint performs flexion and extension around the *X*-axis, adduction and abduction around the *Y*-axis, and internal and external rotations around the *Z*-axis [21].

### 2.4. Data Processing

After performing quality control checks, data from 2 of the 11 subjects were excluded due to missing data and outliers. Then, the coordinates of the marker trajectories from the Vicon system were processed for modeling and gap filling before they were exported as a c3d file to the Visual 3D software for skeletal modeling. Kinetic data and the ground reaction force were normalized to body weight, with inverse dynamics calculation yielding the ankle, knee, and hip joint moments and forces and GRF. The kinematic data included the ankle, knee, and hip joint angles. All biomechanical parameters from the dominant lower limb were time-normalized to 100% of the CMJ duration [22].

Then, the CMJs were divided into six distinct time events based on the vGRF and the velocity of center of mass (vCOM) characteristics [23] (Figure S3). T1 is defined as the time when the vGRF decreases to the threshold value of 5 times the standard deviation of the initial weighing value. T2 is the time when the vCOM reaches the minimum value. The period from T1 to T2 is defined as the unweighting phase (UP). T3 is the time when the vCOM crosses the 0 value after T2. The period from T2 to T3 is defined as the braking phase (BP). T4 is the time when the vGRF decreases to below 10% of the initial weighing value. The period from T3 to T4 is defined as the propulsion phase (PP). T5 is the time when the vGRF exceeds 10% of the initial weighing value. The period from T4 to T5 is defined as the flight phase (FP). T6 is the time when vGRF stabilizes (±5% of body weight). Finally, the period fromT5 to T6 is defined as the landing phase (LP).

### 2.5. Statistical Analysis

SPSS software (version 20.0; IBM Corporation, Armonk, NY, USA) was used for one-way repeated measures analyses of variance (ANOVA) to examine the effect of legwear on CMJ height. Statistical significance was set at *p* < 0.05. Post hoc comparisons with the least significant difference were employed to identify the pairwise differences between conditions. SPM was used to statistically compare the differences in biomechanical characteristics across the conditions. Initially, a repeated measures ANOVA was performed on the normalized time series to identify any significant differences among the three conditions, with post hoc paired *t*-tests used to compare the pairs of conditions. For both the repeated ANOVA and *t*-test analyses, the SPM analysis involved four steps. First, the value of a test statistic at each point in the normalized time series was calculated. Second, temporal smoothness was estimated on the basis of the average temporal gradient. Third, the value of a test statistic was calculated as the value above which only ≥5% of the data would be expected to reach if the test statistic trajectory resulted from an equally smooth random process. Last, the probability that specific suprathreshold regions could have resulted from an equivalently smooth random process was calculated [24]. The technical details are provided in previous publications [25,26]. All SPM analyses were conducted in Matlab (version R2020a; The MathWorks, Inc., Natick, MA, USA) by using the open-source software package spm1D 0.4 (www.spm1d.org) (accessed on 1 Februry 2025) [27].

## 3. Results

### 3.1. Kinematics

As shown in Figure S4, no significant difference was observed for the jump height with different legwear conditions (*p* > 0.05).

**Figure 2:**
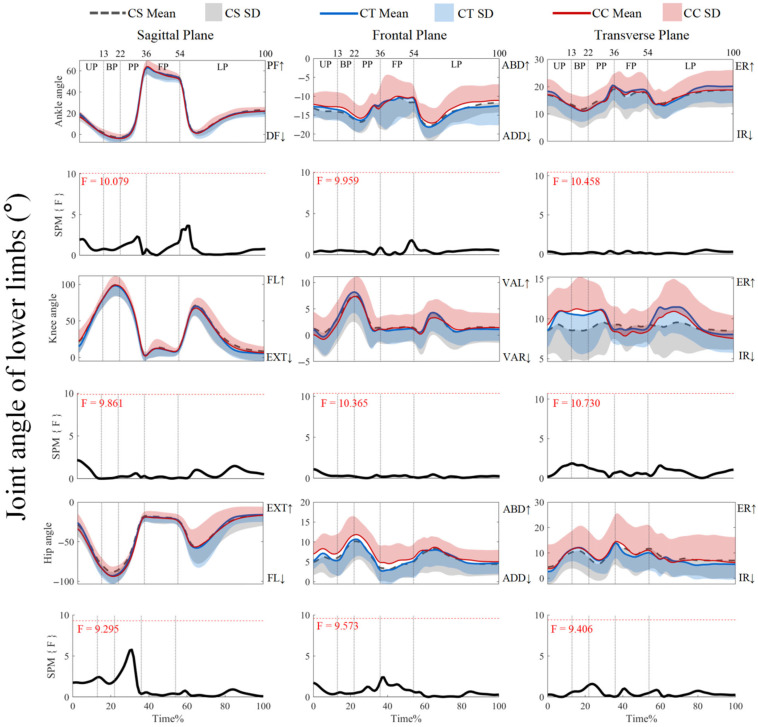
Mean (SD) patterns for lower-limb joint angles with different types of legwear and time-dependent F-values of SPM. UP, unweighting phase. BP, braking phase. PP, propulsion phase. FP, flight phase. LP, landing phase. DF, dorsiflexion. PF, plantarflexion. ADD, adduction. ABD, abduction. IR, internal rotation. ER, external rotation. FL, flexion. EXT, extension. VAR, varus. VAL, valgus. Red dashed line represents the critical threshold. Arrows represents direction of joint motion. Grey dashed line area represents the supra-threshold cluster region.

**Figure 3:**
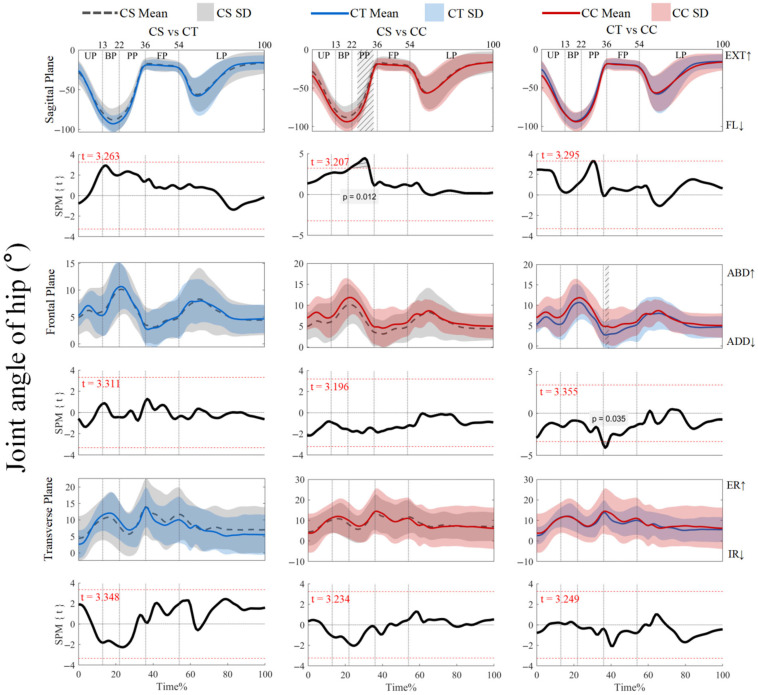
Mean (SD) patterns for hip joint angle with different types of legwear and time-dependent t-values of SPM. UP, unweighting phase. BP, braking phase. PP, propulsion phase. FP, flight phase. LP, landing phase. DF, dorsiflexion. PF, plantarflexion. FL, flexion. EXT, extension. ADD, adduction. ABD, abduction. IR, internal rotation. ER, external rotation. Red dashed line represents the critical threshold. Arrows represent direction of joint motion. Grey dashed line area represents the supra-threshold cluster region.

As shown in Figure 2, the joint angles did not show supra-threshold clusters that exceeded the critical threshold (*p* > 0.05). For ankle joint angles and knee joint angles, post hoc analysis with a paired-*t* test showed that no supra-threshold cluster exceeded the critical threshold (see Figures S5 and S6). As shown in Figure 3, post hoc analysis with a paired-*t* test showed positive supra-threshold clusters (25–36%) exceeding the critical threshold (3.207) as the hip flexion angle with CSs was significantly more positive than with CCs during the PP. Also, the negative supra-threshold clusters (37–39%) exceeded the critical threshold (−3.355) as the hip abduction angle with CTs was significantly more negative than with CCs during FP. Other conditions did not reveal supra-threshold clusters that exceeded the critical threshold.

### 3.2. Kinetics

#### 3.2.1. Joint Moment

**Figure 4:**
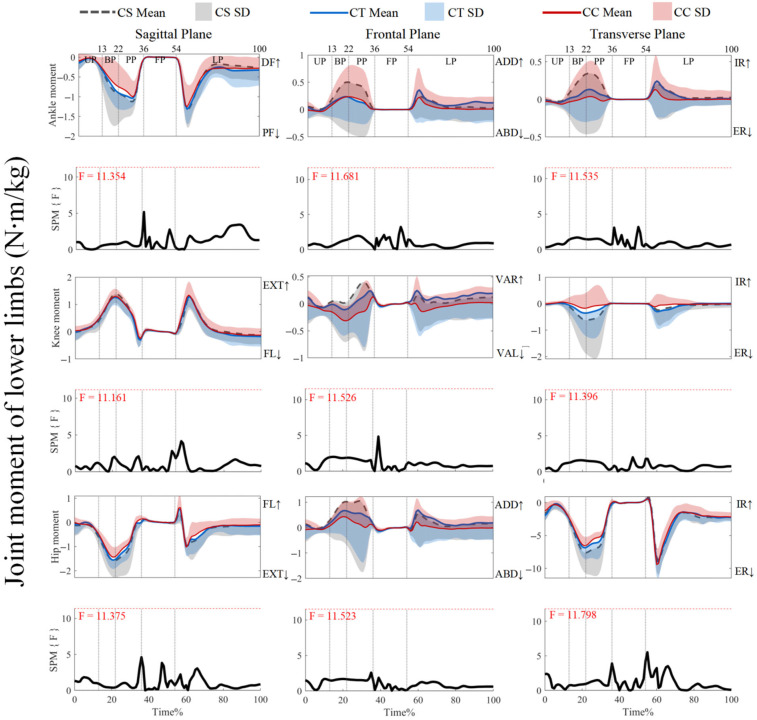
Mean (SD) patterns for lower limb-joint moments with different types of legwear and time-dependent F-values of SPM. UP, unweighting phase. BP, braking phase. PP, propulsion phase. FP, flight phase. LP, landing phase. DF, dorsiflexion. PF, plantarflexion. ADD, adduction. ABD, abduction. IR, internal rotation. ER, external rotation. FL, flexion. EXT, extension. VAR, varus. VAL, valgus. Red dashed line represents the critical threshold. Arrows represent direction of joint motion. Grey dashed line area represents the supra-threshold cluster region.

**Figure 5:**
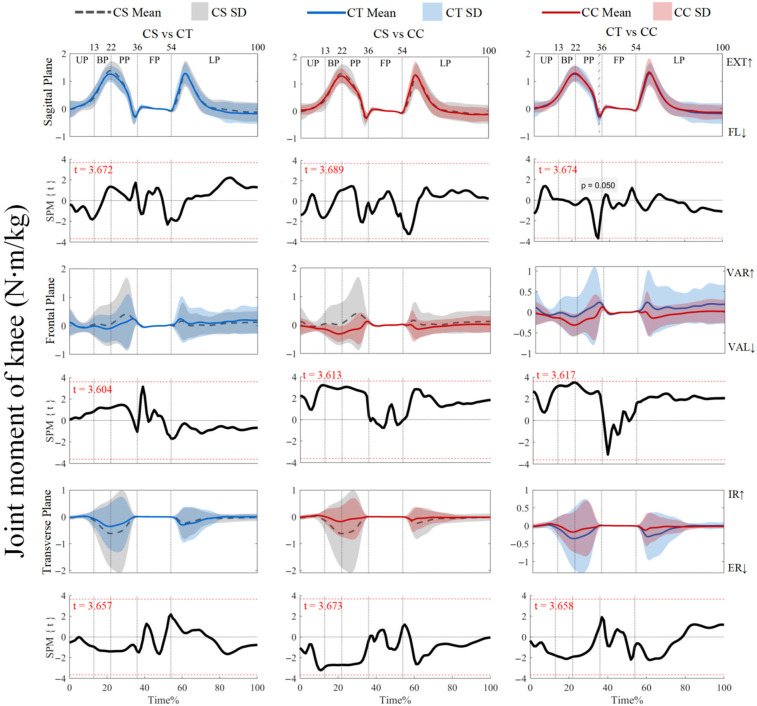
Mean (SD) patterns for knee joint moment with different types of legwear and time-dependent t-values of SPM. UP, unweighting phase. BP, braking phase. PP, propulsion phase. FP, flight phase. LP, landing phase. FL, flexion. EXT, extension. VAR, varus. VAL, valgus. IR, internal rotation. ER, external rotation. Red dashed line represents the critical threshold. Arrows represent direction of joint motion. Grey dashed line area represents the supra-threshold cluster region.

**Figure 6:**
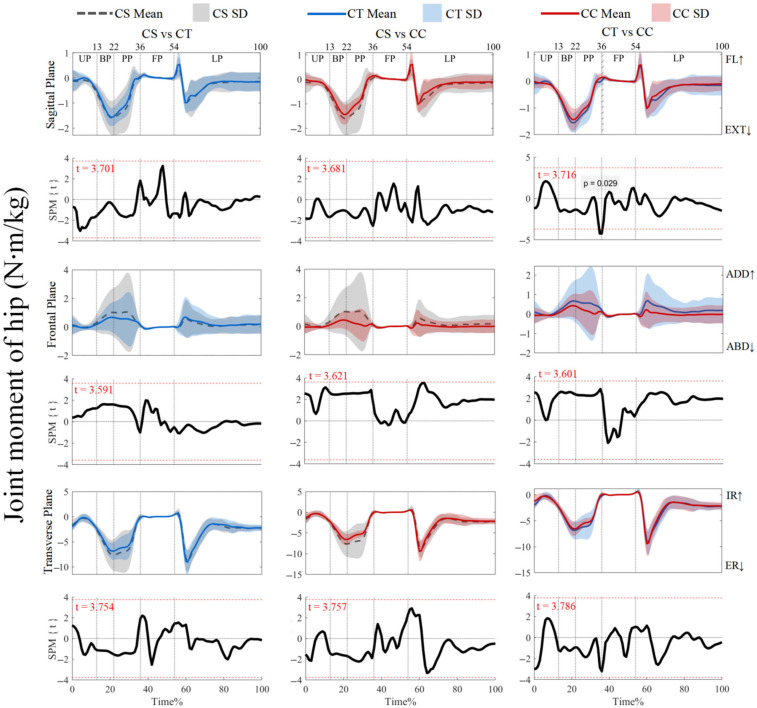
Mean (SD) patterns for hip joint moment with different types of legwear and time-dependent t-values of SPM. UP, unweighting phase. BP, braking phase. PP, propulsion phase. FP, flight phase. LP, landing phase. FL, flexion. EXT, extension. ADD, adduction. ABD, abduction. IR, internal rotation. ER, external rotation. Red dashed line represents the critical threshold. Arrows represent direction of joint motion. Grey dashed line area represents the supra-threshold cluster region.

As shown in Figure 4, the joint moments did not show supra-threshold clusters exceeding the critical threshold (*p* > 0.05). For ankle joint moments, post hoc analysis with a paired-*t* test showed that no supra-threshold cluster exceeded the critical threshold (see Figure S7). However, Figure 5 shows that the post hoc analysis with a paired-*t* test revealed negative supra-threshold clusters (34–35%) exceeding the critical threshold (−3.674), as the knee extension and flexion moments with the use of CTs were significantly more negative than with CCs during PP. Figure 6 also shows that negative supra-threshold clusters (36–37%) exceeded the critical threshold (−3.355), as the hip extension moment with the use of the CTs was significantly more negative than with the use of CCs during FP. The other conditions did not show supra-threshold clusters that exceeded the critical threshold, as shown in Figure 5 and Figure 6.

#### 3.2.2. Joint Forces

**Figure 7:**
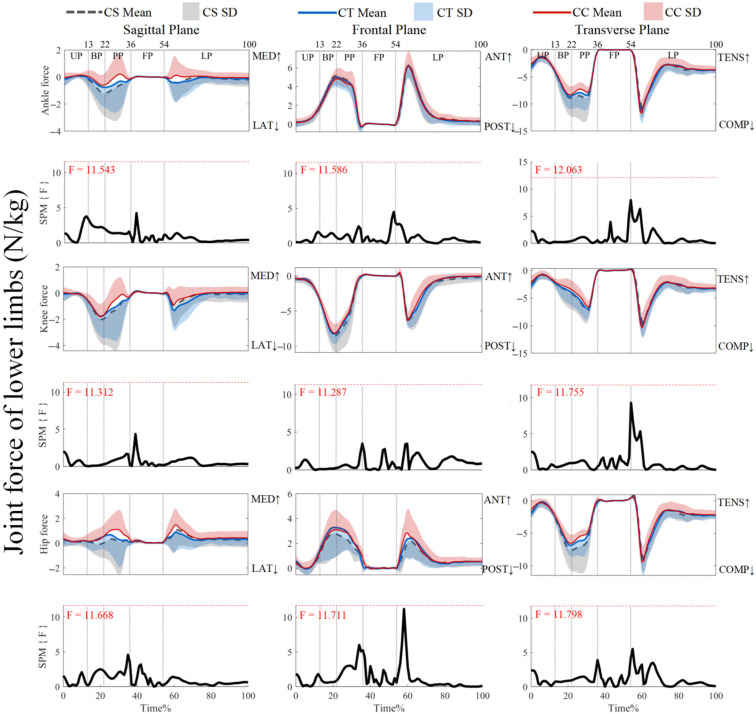
Mean (SD) patterns for lower-limb joint forces with different types of legwear and time-dependent F-values of SPM. UP, unweighting phase. BP, braking phase. PP, propulsion phase. FP, flight phase. LP, landing phase. MED, medial. LAT, lateral ANT, anterior. POST, posterior. TENS, tension. COMP, compression. Red dashed line represents the critical threshold. Arrows represent direction of joint motion. Grey dashed line area represents the supra-threshold cluster region.

**Figure 8:**
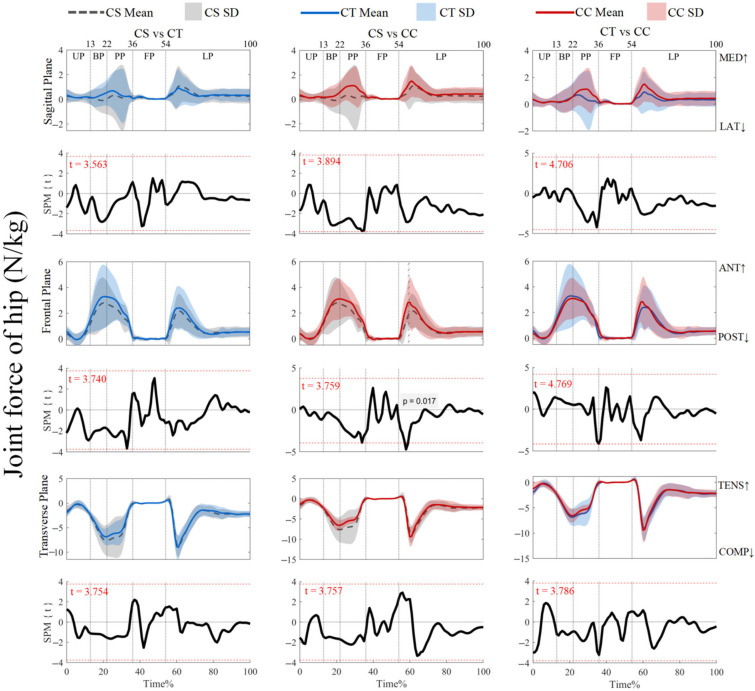
Mean (SD) patterns for hip joint force with different types of legwear and time-dependent t-values of SPM. UP, unweighting phase. BP, braking phase. PP, propulsion phase. FP, flight phase. LP, landing phase. MED, medial. LAT, lateral ANT, anterior. POST, posterior. TENS, tension. COMP, compression. Red dashed line represents the critical threshold. Arrows represent direction of joint motion. Grey dashed line area represents the supra-threshold cluster region.

As shown in Figure 7, the joint forces did not reveal supra-threshold clusters that exceeded the critical threshold (*p* > 0.05). For ankle joint forces and knee joint forces, post hoc analysis with a paired-*t* test showed that no supra-threshold cluster exceeded the critical threshold (see Figures S8 and S9). However, Figure 8 showed that the negative supra-threshold clusters (59–60%) exceeded the critical threshold (−3.759), as the hip force in the anterior direction with the use of CSs was significantly more negative than with the use of CCs during the LP, while no other clusters exceeded the critical threshold.

#### 3.2.3. Vertical Ground Reaction Force

As shown in Figure S10, vGRF did not show supra-threshold clusters that exceeded the critical threshold. In Figure S11, between conditions, it is clear vGRF also did not show any supra-threshold clusters that exceeded the critical threshold in the post hoc analysis with a paired-*t* test.

## 4. Discussion

This study is the first to employ SPM to examine time-continuous biomechanical curves during the CMJ in physically active males wearing three types of legwear. The results revealed that different types of legwear caused distinct biomechanical changes in the lower-limb joints. However, significant differences among the legwear conditions were only detected in the post hoc analysis. In particular, during the PP, the use of CSs significantly reduced the hip flexion angle at (25–36%) compared to the use of CCs, while the use of CTs significantly reduced the knee extension and flexion moment (34–35%) and reduced the hip extension moment (36–37%). During the FP, the use of CT significantly decreased the hip abduction angle compared to the use of CCs (37–39%), and during the LP, the use of CSs significantly reduced the hip anterior force compared to the use of CCs (59–60%). However, there were no other significant biomechanical differences between CSs and CTs, nor any significance in the jump height across the three conditions.

### 4.1. Legwear Effects on Propulsion Phase Biomechanics

The characteristic–time curve of the CMJ is usually divided into six key phases [23], and the joint biomechanical characteristics during the phases prior to take-off mainly influence the jump height [28,29,30]. During the PP (25–36%), CSs significantly reduced the hip flexion angle compared to CCs. This may be due to the pressure that CSs applied to the thigh and hip, which stimulated the mechanoreceptors in the region. This enhanced sensory feedback to the nervous system promoted a neuromuscular response [12,31]. However, it seems that such neurofeedback did not result in measurable enhancements in CMJ performance. Marshall and Moran and Akl [29,32] found that the hip angle at the beginning and end of the PP was significantly correlated with jump height, as full hip extension during the PP helps to raise the center of gravity of the body, which in turn, increases the jump height. In contrast, CSs only significantly changed the hip joint angle during the course of propulsion, and this limited kinematic effect likely contributed to the absence of any increase in jump height with either the CTs or CSs.

During the PP, a larger lower-limb joint moment results in a higher vertical center of mass and an increase in CMJ height [33,34]. CTs significantly reduced the knee extension and flexion moments during the PP compared to CCs. However, the results of the SPM showed a significant difference only at the end of the PP (34–35%), which is consistent with the results of Yu et al. [9]. During jumping tasks, the joint moments are primarily generated by muscles that act on specific joints, which requires efficient activation of the knee-stabilizing musculature [28]. Although higher jump heights are typically associated with larger knee extension moments [30], CTs failed to significantly enhance the jump height in our study. This may be due to its temporary influence on the knee joint moment, which only occurred during a short period of time. Although CTs exerted some pressure on the vastus and Soleus muscles, which are involved in knee extension, the applied pressure was likely below the optimal level [15] and potentially insufficient for efficient neuromuscular adaptation.

### 4.2. Flight and Landing Phases: Implications for Risk of Injury

Neuromuscular output at the hip is also a critical determinant of CMJ performance [35,36]; Shinchi et al. [37] established a strong relationship between the hip extension moment and jump height, as the former directly contributes to increasing the hip and knee angular velocities. Contrary to these established relationships, our findings showed that CTs significantly reduced the hip extension moment at the end of the PP and the start of the FP compared to CCs, with no difference between CTs and CCs observed in propulsion. This kinetic discrepancy likely contributed to the insignificant change in the jump height. On the other hand, Wannop et al. [15] reported that wearing legwear had no significant mechanical effect on the hip joints of athletes, with changes in the hip joint moment dependent on the legwear size, which produced different amounts of applied pressure. In our study, the pressure from CT may have been suboptimal, thus explaining the neutral kinetic outcomes.

Post taking-off, the lower limb biomechanics in the CMJ mainly influence landing performance as well as the risk of injury [38,39,40], as deficits in joint movement coordination in the frontal and sagittal planes may lead to abnormal biomechanical patterns [41]. In Brown et al. [42], the hip abduction angles before the LP typically ranged from 10° to 13°, which is aligned with our findings. The results of the SPM analysis showed that CT significantly reduced the hip abduction angles during the FP compared to CCs. This finding has important clinical implications, as excessive hip abduction prior to landing has been associated with an increased risk of injury to the anterior cruciate ligament (ACL) [41]. Although the region at the suprathreshold is only found at the FP (37–39%), this result suggests that the pressure generated by CTs can be considered as a new biomechanical strategy, which may serve to increase the use of the hip muscles and reduce the loading on the knee in the coronal plane [43]; this may also improve, to some extent, the ability of the hip to align with the knee in the coronal plane, which can reduce the risk of ACL injuries [44].

Attenuating the rapidly increasing impact forces upon ground contact in the LP is critical in real-life sports scenarios, and a mechanical analysis has shown that optimal landing strategies require rapid adaptation to ground reaction forces, in order to not interfere with the subsequent motor tasks [38]. Our results indicated that the use of CSs significantly reduced the anterior hip force during landing compared to the use of CCs. As shown in Figure 8, the region with a significant difference was located at the LP (59–60%), when the hip joint force was peaking, thus indicating that CSs may attenuate the impact force. Optimal landing strategies redistribute the impact so that the knees and ankles absorb the impact [45], and higher peak joint forces are correlated with the increased mechanical work performed at each joint [46]. The observed hip force reduction with the CSs may consequently redistribute the impact absorption demands to other distal joints, thus potentially improving landing efficiency. However, further research is needed to quantify joint-specific contributions.

### 4.3. Limitations

While SPM provides a comprehensive movement analysis by evaluating continuous biomechanical data throughout the entire movement cycle, the post hoc analysis in this study should be interpreted with caution. Further research is needed to clarify the effects of CMJ related to legwear [17,47]. Inter-subject variability in the CMJ kinematics, which is evidenced by the large standard deviations, also poses challenges for the interpretation of SPM. Subsequent studies can be conducted by dividing the subjects into different groups for analysis (e.g., high vs. low CMJ performers), thereby reducing the impact of this variability on the results. Additionally, while the applied pressure values of the compression legwear in this study were based on manufacturer specifications, the actual pressure may vary due to differences in body shape and tissue composition. Future studies should investigate these individual variations.

## 5. Conclusions

The findings of this study suggest that legwear has no significant effect on enhancing CMJ performance. During the PP, CSs significantly reduced the hip flexion angle, while CTs significantly decreased the knee extension and flexion moments, and decreased the hip extension moment during the PP compared to CCs. However, these biomechanical modifications were not substantial enough to improve jump height. Additionally, CTs significantly reduced the hip abduction angle during the FP, and CSs significantly reduced the hip anterior force during the LP. The differences in these parameters suggest that compression legwear may improve landing performance by promoting more favorable joint positioning and force attenuation. Such effects might facilitate safer landings and faster movement transitions in sport-specific scenarios, although the exact mechanisms warrant further investigation.

## Data Availability

The authors declare that the main data and materials that support the findings and conclusions of this study are available within the article.

## Supplementary Materials

The following supporting information can be downloaded at: https://www.mdpi.com/article/10.3390/jfmk10030257/s1. Table S1 Conditions of three types of commercially available compression legwear; Figure S1 Location of reflective markers; Figure S2 Schematic diagram of joints and coordinate systems; Figure S3. Event division of the CMJ; Figure S4. Height of CMJ with different types of legwear; Figure S5. Mean (SD) patterns for ankle joint angle with different legwear and time-dependent t-values of SPM; Figure S6. Mean (SD) patterns for knee joint angle with different legwear and time-dependent t-values of SPM; Figure S7. Mean (SD) patterns for ankle joint moment with different legwear and time-dependent t-values of SPM; Figure S8 Mean (SD) patterns for ankle joint force with different legwear and time-dependent t-values of SPM; Figure S9 Mean (SD) patterns for knee joint force with different legwear and time-dependent t-values of SPM; Figure S10. Mean (SD) patterns for vertical ground reaction forces with different legwear and time-dependent F-values of SPM; Figure S11; Mean (SD) patterns for vertical ground reaction force with different legwear and time-dependent t-values of SPM.

## Abbreviations

The following abbreviations are used in this manuscript:

CMJ	Countermovement jump
CG	Compression garment
GRF	Ground reaction force
CT	Compression tight
CS	Compression short
CC	Control short
SPM	Statistical parametric mapping
vCOM	Velocity of center of mass
HAMS	Hamstring
SOL	Soleus
VAS	Vastus
GMAX	Gluteus maximus
GAS	Gastrocnemius
UP	Unweighting phase
BP	Braking phase
PP	Propulsion phase
FP	Flight phase
LP	Landing phase
DF	Dorsiflexion
PF	Plantarflexion
ABD	Abduction
ADD	Adduction
IR	Internal rotation
ER	External rotation
FL	Flexion
EXT	Extension
VAR	Varus
VAL	Valgus
MED	Medial
LAT	Lateral
ANT	Anterior
POST	Posterior
COMP	Compression
TENS	Tension
