# Supplementary material for: Acute Effects of Different Types of Compression Legwear on Biomechanics of Countermovement Jump: A Statistical Parametric Mapping Analysis
Source: J Funct Morphol Kinesiol. 2025 Jul 7;10(3):257. doi: 10.3390/jfmk10030257 (PMC12286225; doi:10.3390/jfmk10030257)
Supplement: Supplementary file 1 [file jfmk-10-00257-s001.zip › jfmk-3731161-supplementary.pdf]

Supplementary Materials

Table S1. Conditions of three types of commercially available compression legwear

Condition	Length	Material	Compression amount
CC	Above-knee	100% spandex	0-5 mmHg
CS	Above-knee	72% nylon, 28% Lycra in front<br>65% nylon, 35% Lycra in back	20-30 mmHg
CT	Ankle	72% nylon, 28% Lycra in front<br>65% nylon, 35% Lycra in back	23-25 mmHg

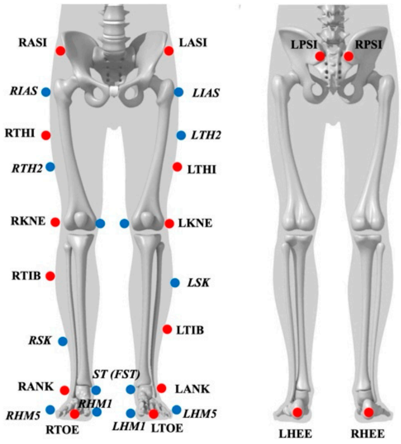

Figure S1. Location of reflective markers

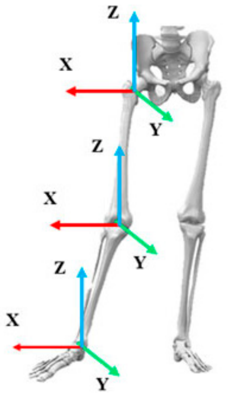

Figure S2. Schematic diagram of joints and coordinate systems.

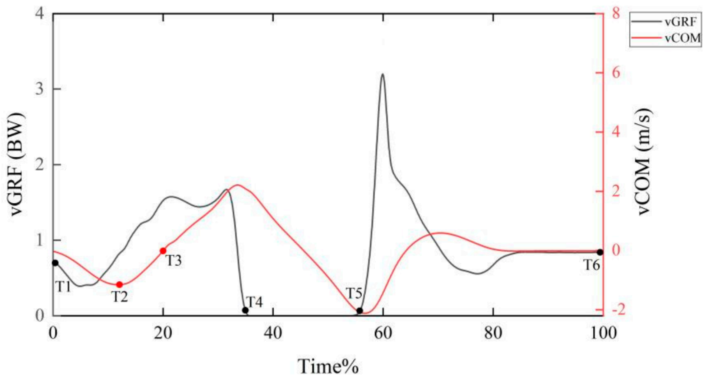

Figure S3. Event division of the CMJ

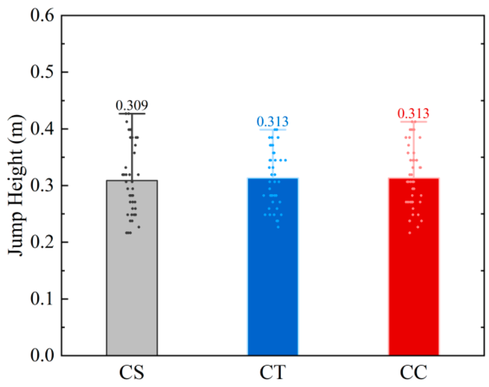

Figure S4. Height of CMJ with different types of legwear

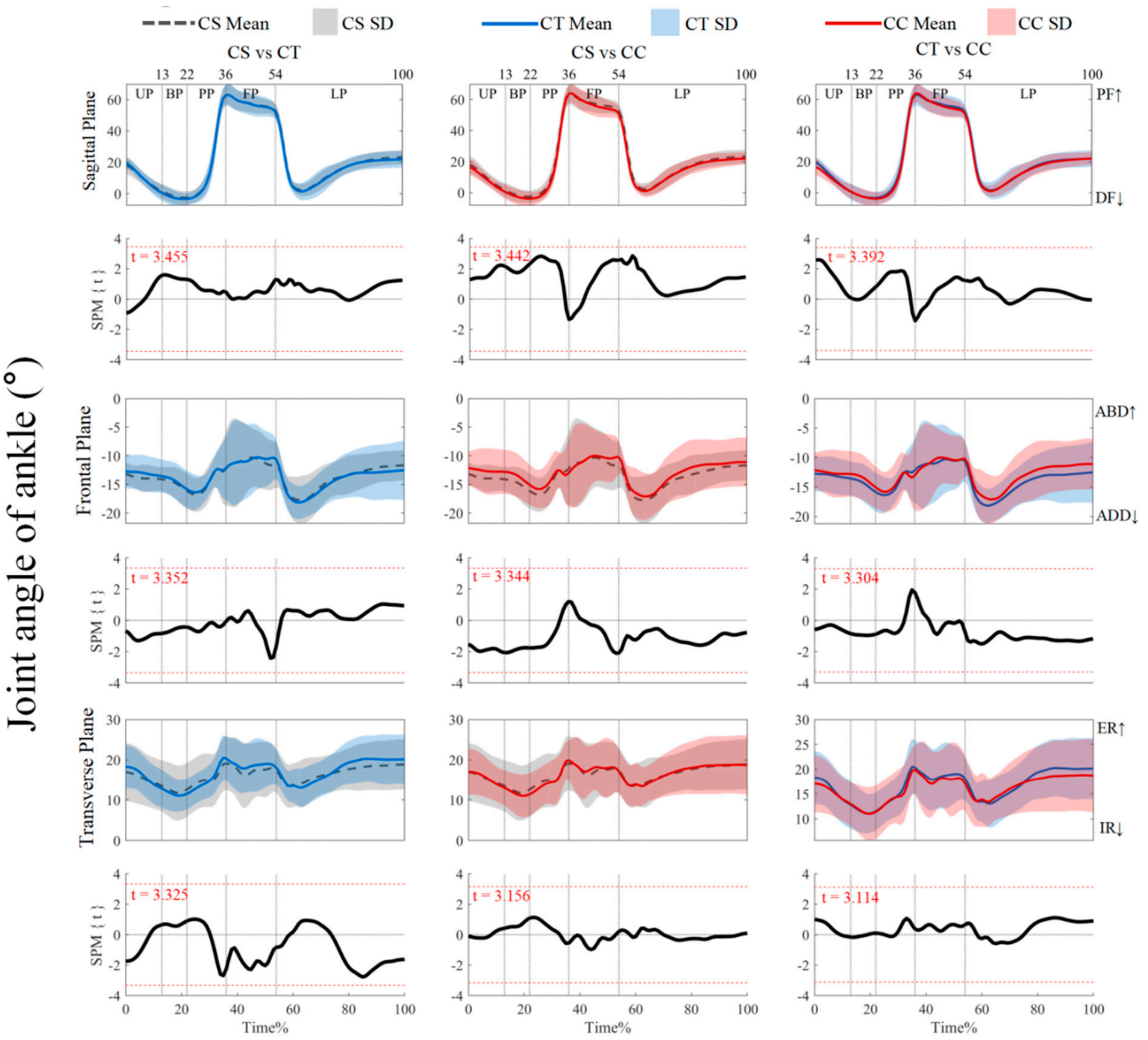

Figure S5. Mean (SD) patterns for ankle joint angle with different legwear and time-dependent t-values of SPM.–UP, unweighting phase. BP, braking phase. PP, propulsion phase. FP, flight phase. LP, landing phase. DF, dorsiflexion. PF, plantarflexion. ADD, adduction. ABD, abduction. IR, internal rotation. ER, external rotation. Red dashed line represents the critical threshold. Arrows represents direction of joint motion.

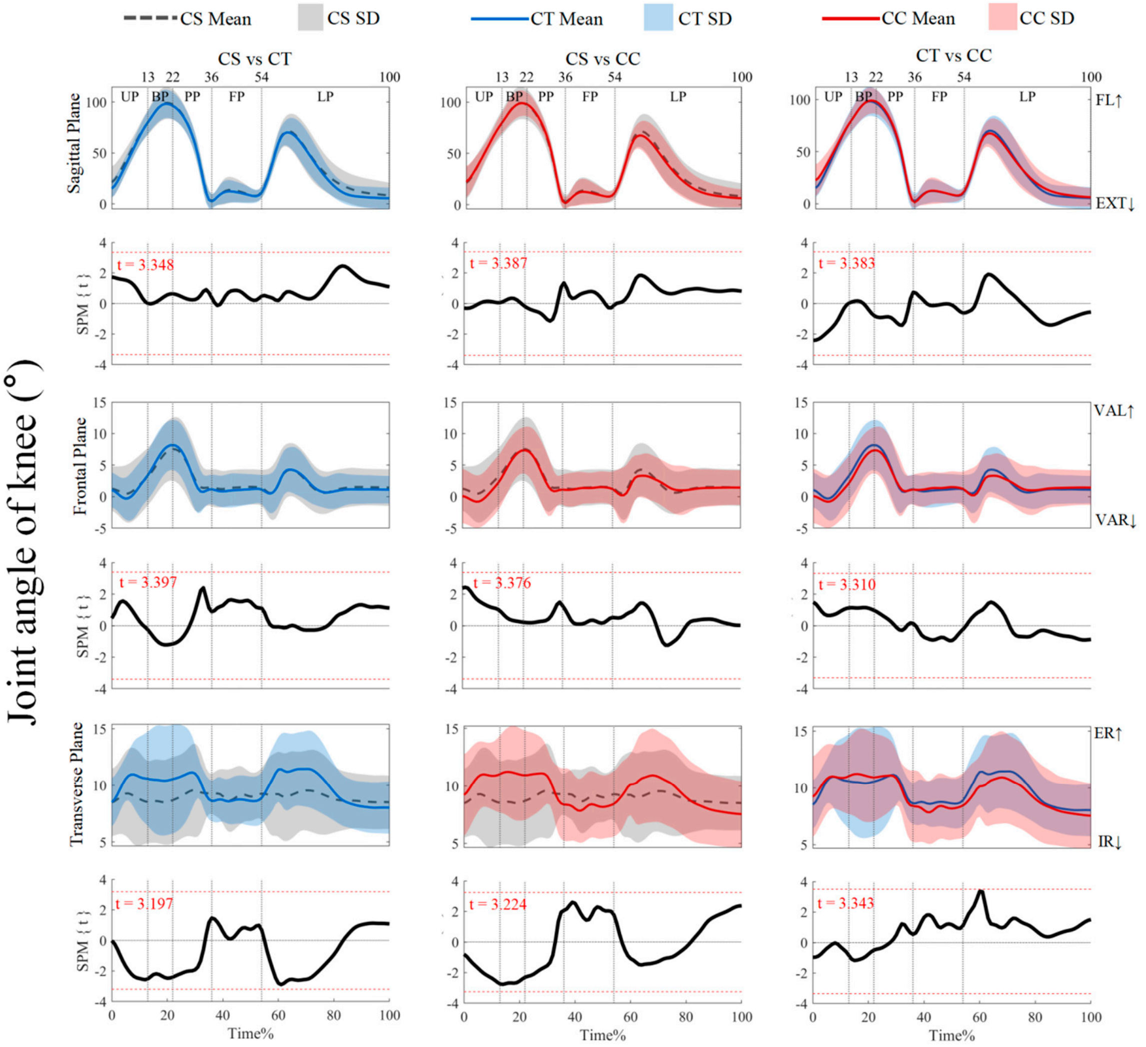

Figure S6. Mean (SD) patterns for knee joint angle with different legwear and time-dependent t-values of SPM. UP, unweighting phase. BP, braking phase. PP, propulsion phase. FP, flight phase. LP, landing phase. FL, flexion. EXT, extension. VAR, varus. VAL, valgus. IR, internal rotation. ER, external rotation. Red dashed line represents the critical threshold. Arrows represents direction of joint motion.

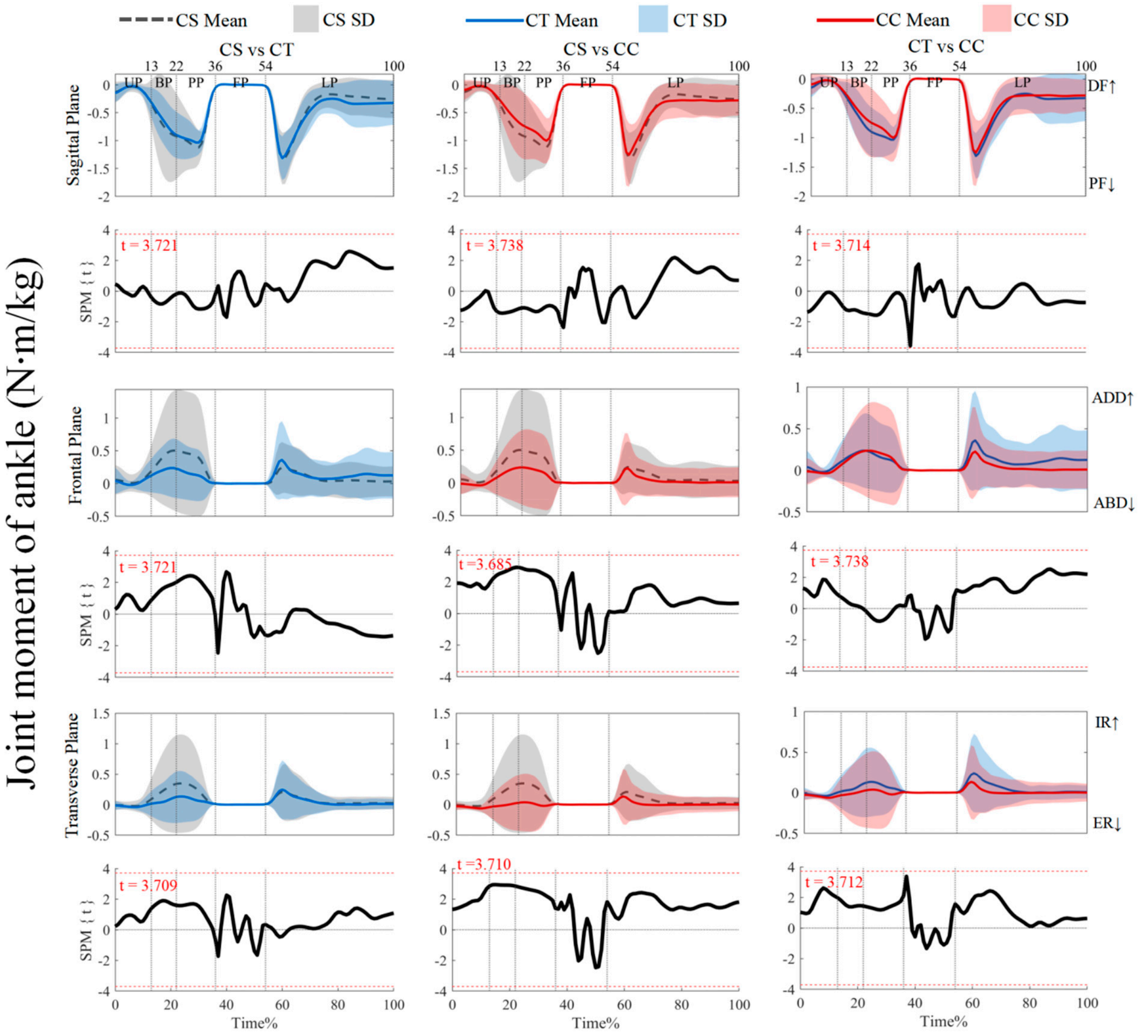

Figure S7. Mean (SD) patterns for ankle joint moment with different legwear and time-dependent t-values of SPM. UP, unweighting phase. BP, braking phase. PP, propulsion phase. FP, flight phase. LP, landing phase. DF, dorsiflexion. PF, plantarflexion. ADD, adduction. ABD, abduction. IR, internal rotation. ER, external rotation. Red dashed line represents the critical threshold. Arrows represents direction of joint motion.

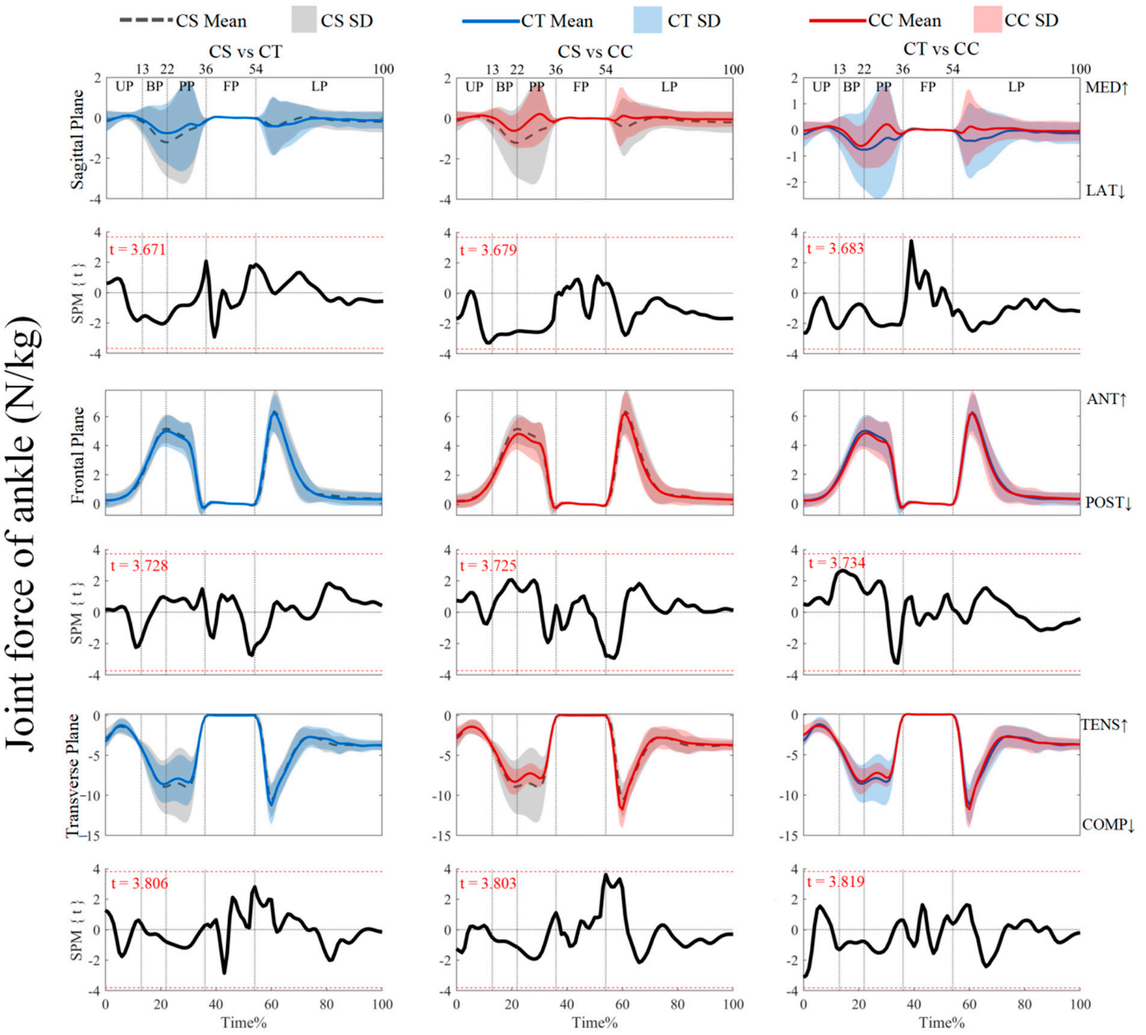

Figure S8. Mean (SD) patterns for ankle joint force with different legwear and time-dependent t-values of SPM. UP, unweighting phase. BP, braking phase. PP, propulsion phase. FP, flight phase. LP, landing phase. MED, medial. LAT, lateral ANT, anterior. POST, posterior. TENS, tension. COMP, compression. Red dashed line represents the critical threshold. Arrows represents direction of joint motion.

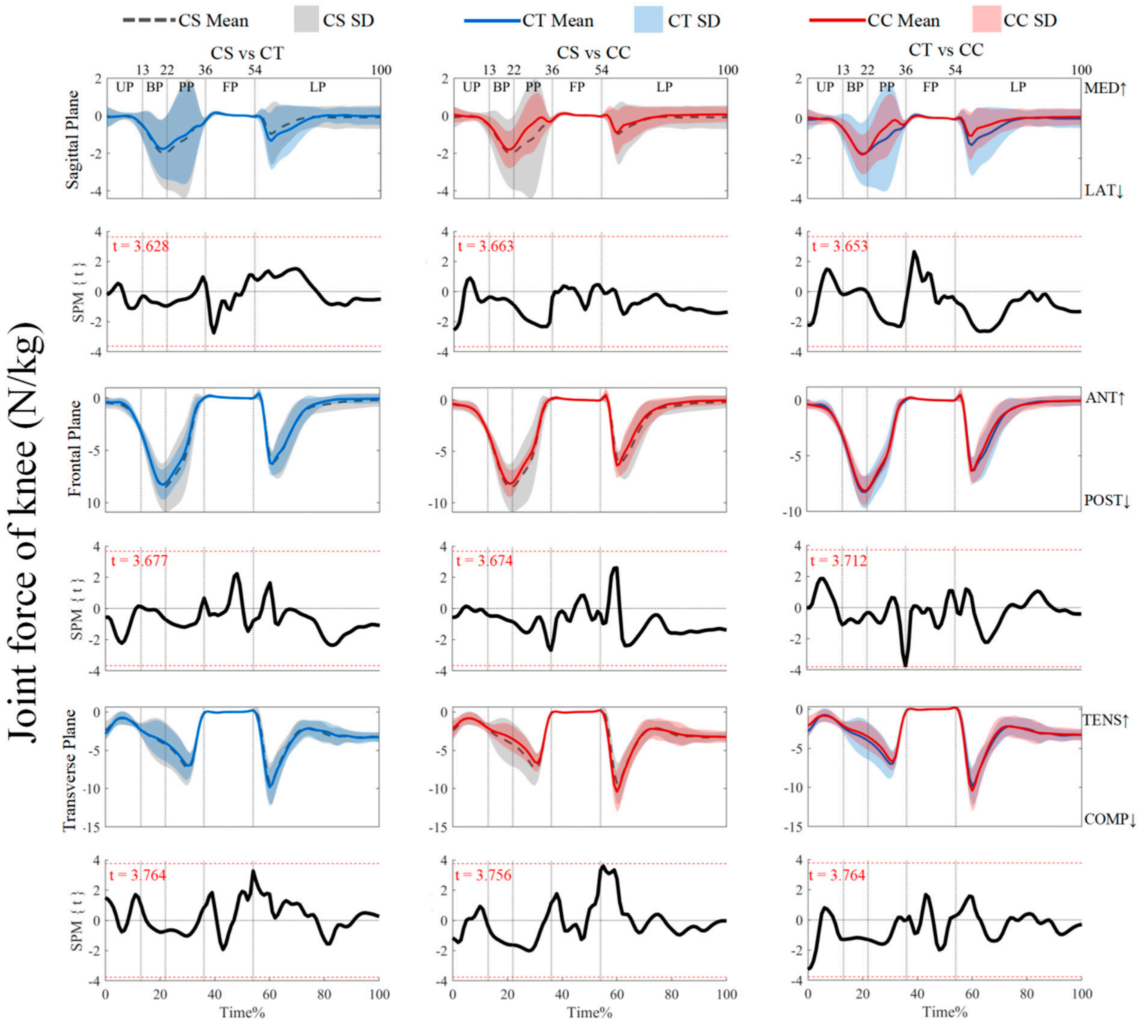

Figure S9. Mean (SD) patterns for knee joint force with different legwear and time-dependent t-values of SPM. UP, unweighting phase. BP, braking phase. PP, propulsion phase. FP, flight phase. LP, landing phase. MED, medial. LAT, lateral ANT, anterior. POST, posterior. TENS, tension. COMP, compression. Red dashed line represents the critical threshold. Arrows represents direction of joint motion.

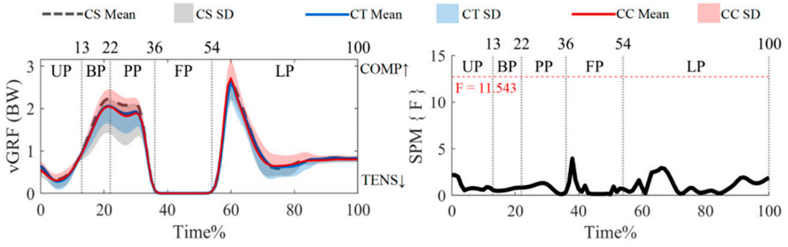

Figure S10. Mean (SD) patterns for vertical ground reaction forces with different legwear and time-dependent F-values of SPM. UP, unweighting phase. BP, braking phase. PP, propulsion phase. FP, flight phase. LP, landing phase. TENS, tension. COMP, compression. Red dashed line represents the critical threshold. Arrows represents direction of joint motion.

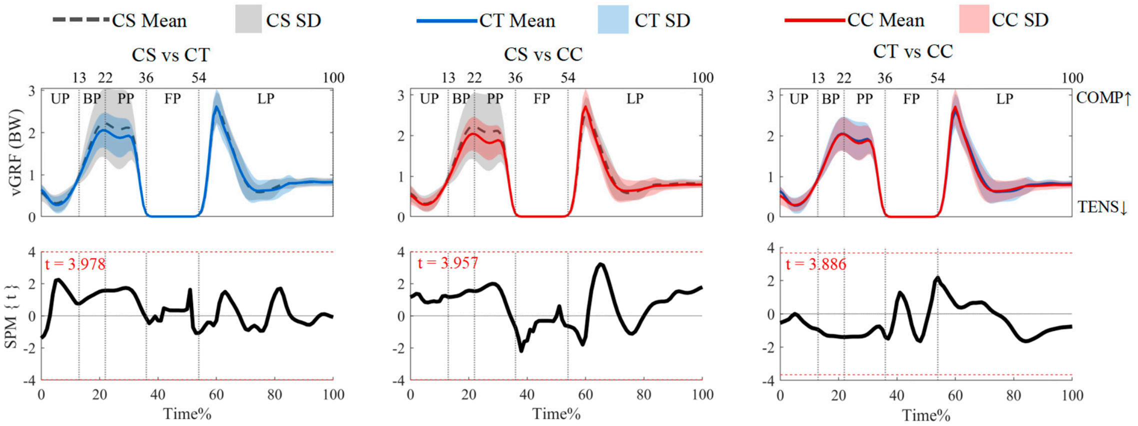

Figure S11. Mean (SD) patterns for vertical ground reaction force with different legwear and time-dependent t-values of SPM. UP, unweighting phase. BP, braking phase. PP, propulsion phase. FP, flight phase. LP, landing phase. TENS, tension. COMP, compression. Red dashed line represents the critical threshold. Arrows represents direction of joint motion.
